# Successful two-stage operation for esophageal necrosis due to proton beam therapy followed by sorafenib in a case of large hepatocellular carcinoma

**DOI:** 10.1186/s40792-020-00902-0

**Published:** 2020-06-16

**Authors:** Eiji Higaki, Masahide Fukaya, Kazushi Miyata, Ryosuke Kawai, Tetsuya Abe

**Affiliations:** 1 Division of Surgical Oncology, Department of Surgery, Nagoya University Graduate School of Medicine, 65 Tsurumai-cho, Showa-Ku, Nagoya, Aichi 466-8550 Japan; 2 Department of Gastroenterological Surgery, Aichi Cancer Center Hospital, 1-1 Kanokoden, Chikusa-ku, Nagoya, Aichi 464-8681 Japan

**Keywords:** Hepatocellular carcinoma, Necrosis, Esophagus, Sorafenib

## Abstract

**Background:**

Locally advanced hepatocellular carcinoma (HCC), which is unsuitable for standard locoregional therapies, remains a challenge to manage. Among the recently developed treatments, proton beam therapy (PBT) has been reported to achieve good local control. However, in patients with large HCC adjacent to the esophagus, high-dose PBT may rarely lead to radiation-induced esophageal necrosis or perforation. Furthermore, the optimal strategy to safely treat these fatal complications remains unclear.

**Case presentation:**

A 49-year-old man who was diagnosed with a large (16 cm) HCC in the right lobe with tumor thrombosis in the main trunk of the portal vein (PVTT) received high-dose hypofractionated PBT in another hospital. A total dose of 66 GyE in 10 fractions was administered to the primary tumor and the PVTT. After 5 months, a 1-cm solitary nodule was noted in the upper lobe of the right lung. Therefore, sorafenib was started. About 6 months after the PBT, lower esophageal mucosal inflammation that progressed to an ulcer was noted. About 7 months after the PBT, the lower esophagus developed full-thickness necrosis. Therefore, emergency thoracoscopic esophagectomy was performed, followed by two-stage reconstruction 2 months later. The operation and postoperative clinical course were mostly uneventful, except for a minor anastomotic leakage. The outcome of the primary HCC, including the PVTT, was graded as a complete response, which has been maintained for 51 months after the PBT.

**Conclusion:**

PBT is a promising option for patients with locally advanced HCC; however, for large tumors adjacent to the esophagus, ischemic esophageal necrosis due to antiangiogenic effects may occur, particularly with the combined use of PBT and sorafenib. In such a life-threatening condition, the thoracoscopic esophagectomy and the two-stage reconstruction are a safe option that can prevent critical postoperative complications due to the poor general condition, effects of PBT on the remnant gastric conduit, and use of sorafenib.

## Introduction

Hepatocellular carcinoma (HCC) is one of the most common cancers in the world and is especially prevalent in Asia and Africa. In contrast to other cancers, HCC may be treated with various modalities, such as surgical resection, liver transplantation, transcatheter arterial chemoembolization, radiofrequency ablation, sorafenib, and external beam radiation therapy (ERBT), as enlisted in the current clinical guidelines [1–3]. Despite the recent progress in these HCC treatments, strategies for locally advanced tumors that are unsuitable for standard locoregional therapies remain a challenging area.

Proton beam therapy (PBT) is a type of ERBT that uses charged particles and can increase energy deposition at a penetration depth of up to a sharp maximum to form the so-called Bragg peak [4]. Compared with standard photon beams, PBT can distribute tumoricidal doses without aggravating toxicity in the adjacent normal organs. Therefore, PBT has been reported to achieve good local control and less toxicities for patients with locally advanced HCC, such as those presenting as large tumors [5] and those with portal vein tumor thrombosis (PVTT) [6] or inferior vena cava tumor thrombus [7]. However, in patients with an HCC adjacent to the gastrointestinal (GI) tract, a high dose of PBT may cause radiation-induced ischemic bowel complications or bowel perforation. In addition, a combination of radiotherapy (RT) and sorafenib, which is a potent multikinase inhibitor with antiangiogenic and antiproliferative properties, may result in a higher risk for serious bowel complications secondary to delayed microvasculature recovery, compared to that expected with either treatment alone [8].

This study reported a case of radiation-induced esophageal necrosis after complete response in a patient with large HCC involving PVTT in the main trunk who was treated with PBT followed by sorafenib; the case was successfully treated via thoracoscopic esophagectomy and two-stage reconstruction.

## Case presentation

**Fig. 1:**
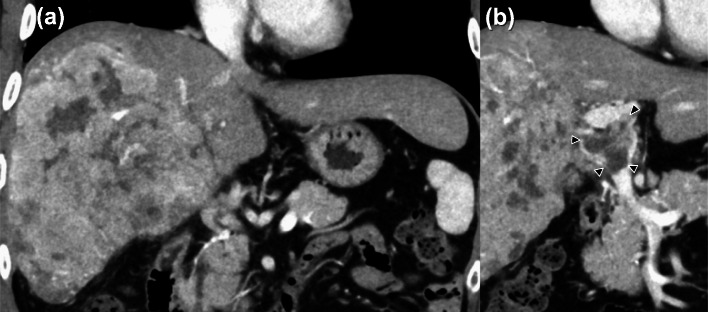
CT findings before proton beam therapy. **a** A hepatic tumor with a diameter of 16.0 cm is observed in the right lobe. **b** The tumor is accompanied by portal vein tumor thrombosis in the main trunk (black arrowheads)

**Fig. 2:**
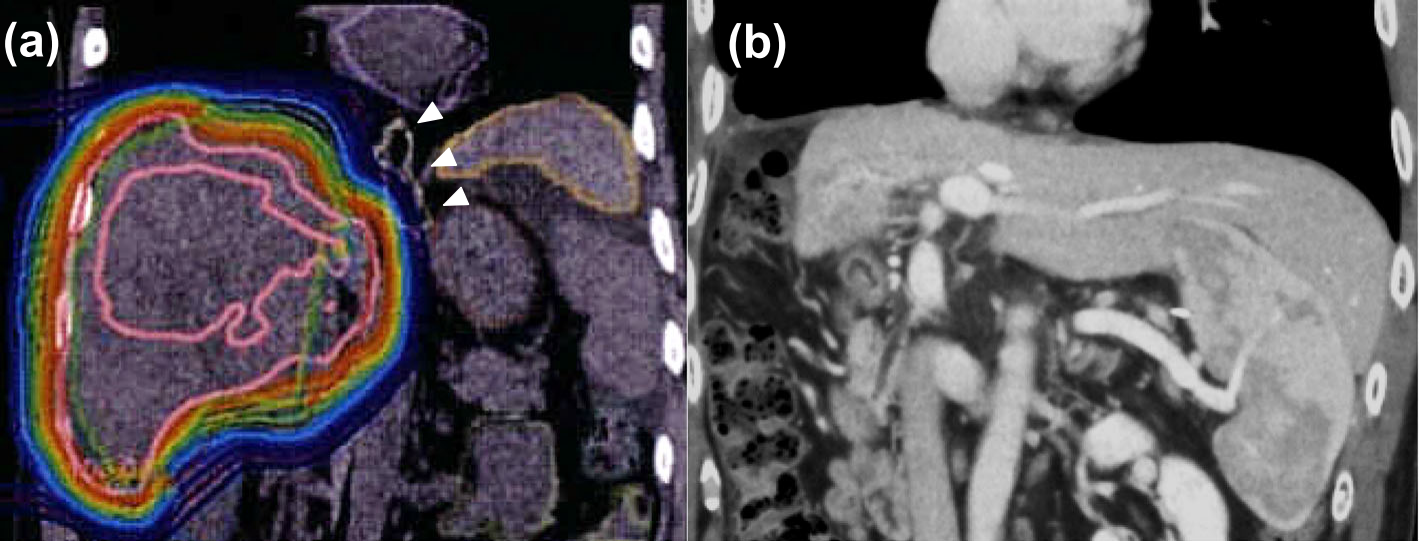
Dose distribution during proton beam therapy planning and CT findings after the therapy. **a** Dose distribution during proton beam therapy planning. The white arrowheads indicate the esophagus. **b** At 5 months after the administration of 66 GyE proton beam therapy in 10 fractions on the primary hepatocellular carcinoma with portal vein tumor thrombosis in the main trunk, CT was done, and the outcome was graded as a complete response, according to the RECIST

A 49-year-old man was diagnosed with a 16-cm bulky HCC in the right lobe (Fig. 1a) with PVTT in the main trunk (Fig. 1b). We attempted curative surgical resection initially, but collateral vessels in the hepatoduodenal ligament were noted intraoperatively. Substantial hemorrhage was assumed; therefore, the treatment for the primary tumor and the PVTT was converted to high-dose hypofractionated PBT for a total dose of 66 GyE in 10 fractions, which were administered in another hospital (Fig. 2a). There was no acute toxicity exceeding grade 1. After 5 months of PBT completion, the outcome of the primary HCC, including the PVTT, was graded as a complete response (CR), according to the response evaluation criteria in solid tumors (Fig. 2b). However, a 1-cm solitary pulmonary nodule was noted in the right upper lobe on computed tomography (CT). Therefore, daily oral sorafenib (800 mg) was administered as molecular-targeted drug therapy.

**Fig. 3:**
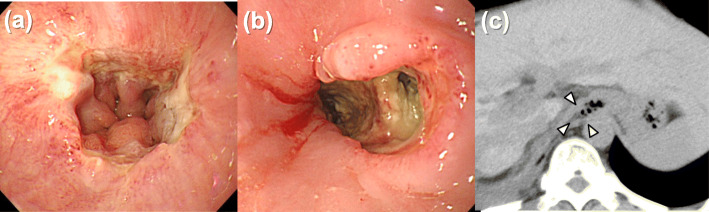
Time series of the esophageal findings on upper endoscopy and CT**. a** After 6 months of the PBT, mucosal inflammation is noted in the lower esophagus. After 7 months of the PBT, **b** there is worsening of the esophageal ulcer into full-thickness necrosis, and **c** CT reveals a wall defect on the right side of the lower esophagus (white arrowheads). CT, computed tomography; PBT, proton beam therapy

About a month after the initiation of sorafenib, the patient complained of heartburn. Upper endoscopy revealed mucosal inflammation in the lower esophagus (Fig. 3a), and a proton-pump inhibitor was started for reflux esophagitis. After 1 more month, the esophageal lesion progressed to a severe ulcer. After hospitalization, sorafenib was discontinued, and the patient was placed on nothing per orem. On day 7 after the admission, the esophageal ulcer progressed to full-thickness necrosis (Fig. 3b), and CT revealed a wall defect on the right side of the lower esophagus (Fig. 3c). Based on these findings, the patient was diagnosed as necrotic perforation of the lower esophagus.

**Fig. 4:**
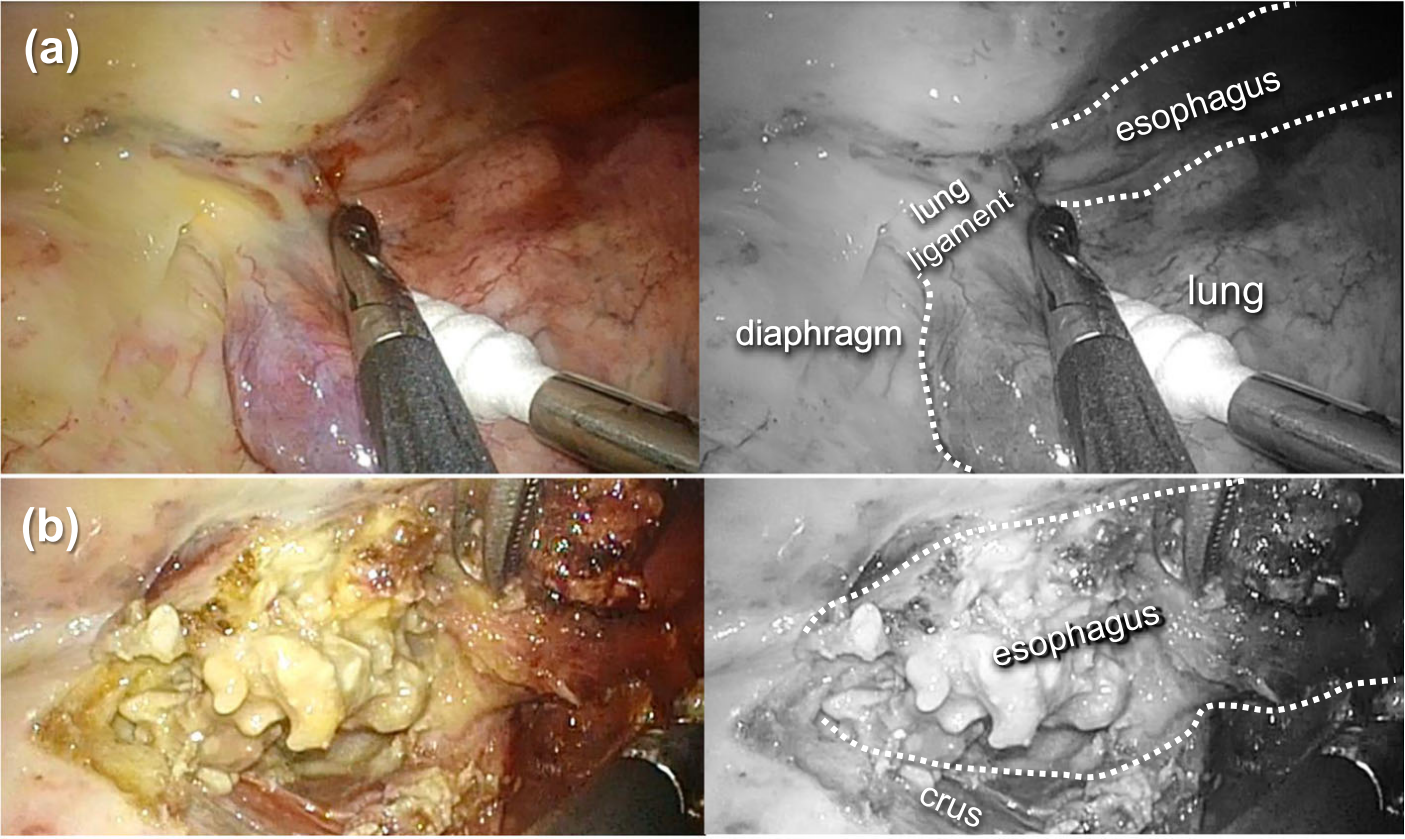
Intraoperative findings. **a** The right lower mediastinal pleura and diaphragm around the irradiation field of proton beam therapy show ischemic changes with thickened wall in yellowish-white color. **b** The right lower esophageal wall above the diaphragm is necrosed in its full thickness

**Fig. 5:**
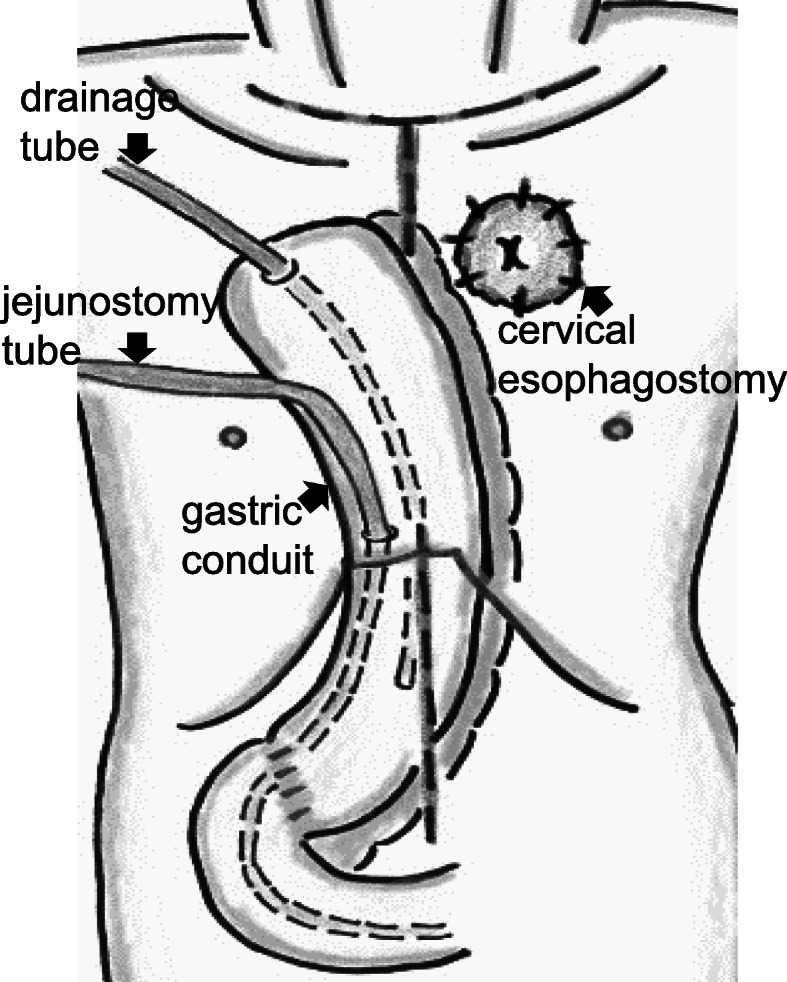
Postoperative schema. The gastric conduit is pulled up and through the subcutaneous route, followed by creation of cervical esophagostomy. A gastrostomy tube for drainage and a jejunostomy tube for postoperative enteral nutrition are placed on the subcutaneous gastric conduit

He underwent emergency thoracoscopic esophagectomy in a prone position. Intraoperatively, the right lower mediastinal pleura and diaphragm around the irradiation field were noted to have thickened and developed a yellowish-white color, indicating ischemic changes (Fig. 4a). The right pleural cavity contained serous pleural effusion. The right lower esophageal wall above the diaphragm showed full-thickness hypovascular necrosis (Fig 4b), and both crura of the diaphragm were inflamed and strongly adherent to the esophagus. We performed subtotal esophagectomy combined with partial diaphragmatic crus resection. Since the necrotic changes in the serosa extended into the gastric cardia, simultaneous resection of the upper stomach was unavoidable. A gastric conduit was made using the macroscopically intact region and was pulled up and through the subcutaneous route. We decided to avoid performing simultaneous esophagogastrostomy, considering the poor general condition, the effect of PBT to the remnant stomach, and the sorafenib use. Finally, external esophagostomy was created in the neck, followed by placement of a gastrostomy tube for drainage and a jejunostomy tube for postoperative enteral nutrition on the subcutaneous gastric conduit. The postoperative schema is shown in Fig. 5.

Two months after the first surgery, anastomosis of the remnant cervical esophagus and the gastric conduit was performed as a two-stage operation. Minimal anastomotic leakage was observed on postoperative day (POD) 18 and was successfully managed by conservative therapy. The patient was discharged on POD 44 without other complications.

To date, the HCC with PVTT has been maintained on prolonged CR for 51 months after the PBT, and the pulmonary nodule has remained stable.

## Discussion

We presented a case of bulky HCC with PVTT in the main trunk that achieved local CR after hypofractionated PBT. The National Comprehensive Cancer Network guidelines of 2017 suggest that PBT may be appropriate in specific situations of locoregional therapy; however, treatment at centers with experience is recommended [3]. PBT may be considered appropriate in cases with unresectable, locally advanced, or recurrent HCC. Recently, hypofractionated RT in approximately 10 fractions has been attempted to reduce treatment duration, give more convenience for patients, and increase the biologic effect of RT. Hong et al. demonstrated that high-dose hypofractionated PBT for HCC achieved a high local control rate of 94.8% at 2 years and less toxicity rate of 2% [9]. Several studies are ongoing to investigate the impact of hypofractionated PBT on HCC outcomes.

PBT-induced toxicity to the adjacent normal GI tract was reported to be a rare complication; nevertheless, serious problems should be considered with caution [10]. The late toxicities of PBT to an adjacent GI tract are caused by local micro ischemia, which can occur gradually but is progressive; it may frequently occur after more than half a year after PBT.

There were several reasons that may have caused the esophageal necrosis in our case. First, the actual irradiation may not have matched the planned dose distribution, especially for the lesion in contact with the diaphragm, owing to respiratory motion. With the planned dose distribution in our case, the esophagus should have been hardly irradiated. However, because the proton beam from the horizontal irradiation port encompassed the nearby right lower lobe of the lung on the level of the esophagogastric junction, a large respiratory motion might have displaced the actual irradiation from the planned position of distal fall and Bragg peak. Second, the administration of sorafenib at 5 months after the PBT completion might have aggravated the esophageal necrosis. Sorafenib, a multikinase inhibitor, has activities against Raf kinase and several receptor tyrosine kinases, such as vascular endothelial growth factor receptor 2, platelet-derived growth factor receptor, FLT3, Ret, and c-Kit, and inhibits tumor angiogenesis in HCC [11]. A phase II trial showed that RT combined with sorafenib therapy achieved 55% complete or partial response rate in patients with unresectable HCC [12]. Theoretically, the combination therapy is a promising strategy and is expected to exert synergistic antiangiogenesis effects; however, its treatment-related toxicity has not been formally evaluated. The vascular endothelial growth factor signaling pathway is needed for the recovery of PBT-induced microvasculature damage in normal organs, but this pathway is blocked in patients receiving sorafenib after PBT. Barney et al. reported that 9% of patients who received vascular endothelial growth factor receptor inhibitor after stereotactic body radiation therapy in 3–5 fractions developed grade 3–5 bowel complications, such as ulcer or perforation [8]. In our case, even if the proton dose applied to the esophagus was low, the occurrence of ischemic esophageal necrosis remained possible because of the synergistic antiangiogenic effects of sorafenib and PBT. On the other hand, it is noteworthy that Chen et al. demonstrated only one case (2.8%) of > grade 3 bowel complication after combined sorafenib therapy and RT in a conventional fraction size ranging from 2.0 to 2.5 Gy [12]. Compared with hypofractionated PBT, the use of conventional fraction size during PBT may reduce the risk for ischemic bowel complications in cases of large HCC adjacent to the GI tract, like our case.

Esophageal necrosis or perforation is a life-threatening condition. In this case, we safely completed the thoracoscopic esophagectomy and the two-stage reconstruction for the extensive radiation-induced necrosis, which spread from the lower esophagus to the upper stomach. There have been only few studies regarding the use of thoracoscopic treatment in such emergency settings. Recently, the safety and efficacy of thoracoscopic management for severe spontaneous esophageal perforation were reported [13]. In the presence of strong adhesions, fibrotic changes of the tissues or obscuration of the anatomic layer structures are caused by radiation therapies as seen in this case, and magnification of thoracoscopy may help surgeons to identify the dissection line between the fibrotic tissue and the adjacent normal organ (Supplementary Fig. S1). A further study is required to determine the efficacy of the thoracoscopic approach for critical esophageal illness. The transhiatal approach should be considered in terms of minimally invasiveness, but in our case, with wide spreading local necrosis and strong adhesion, transthoracic approach would be suitable than transhiatal esophagectomy which includes blind and blunt dissections.

Two-stage esophagectomy has been reported as a safe option that can prevent critical postoperative complications in patients with esophageal cancer at high risk [14]. However, there is insufficient evidence regarding its use in benign diseases, including esophageal necrosis. Prolonged hospital stay, mental stress, and economic burden are disadvantage of the procedure; therefore, a reasonable indication is necessary. In this case, two-stage operation was suitable for two reasons: First, septic risk due to esophageal necrosis and anastomotic risk due to the severe antiangiogenesis effect of PBT combined with sorafenib could be separately managed by two-stage operation. Second, some length of time was needed to observe whether the gastric conduit created showed further progression of ischemia. Gastric conduit is not generally created in the first stage of two-stage operation, and only esophagectomy, external esophagostomy, and gastrostomy are performed. In this case, esophagectomy and the creation of a gastric conduit were performed at the same time in the first operation since the gastric cardia could not be divided due to PBT-induced damage of the upper stomach, and proximal gastrectomy was unavoidable. The gastric conduit was subsequently pulled up through the subcutaneous route to be able to deal with further progression of ischemia. Esophagogastrostomy was performed in the second operation after confirming the stability of the gastric conduit. Our modified two-stage operation may be beneficial when stomach damaged by radiation therapy is intended to be used for reconstruction.

## Conclusions

PBT is one promising option in patients with locally advanced HCC, but for large tumors adjacent to the GI tract, it would be necessary to pay careful attention to accurate radiation planning to avoid radiation-induced bowel complications. Sorafenib administration after PBT for HCC adjacent to the GI tract may inhibit microvasculature recovery and can lead to irreparable RT-related bowel complications. Further improvements of PBT are warranted, and patients should be carefully monitored for the development of late-phase GI complications.

Additionally, in such a life-threatening condition, the thoracoscopic esophagectomy and two-stage reconstruction are a safe option that can prevent critical postoperative complications due to poor general condition, effects of PBT to the remnant gastric conduit, and use of sorafenib.

## Supplementary information

**Additional file 1.** Firstly, the operation was started from the normal anatomical region(a) and then the dissection line was connected to the fibrotic region(b), confirming the adjacent normal anatomy with magnified view(c).

## Data Availability

Not applicable.

## Abbreviations

HCC: Hepatocellular carcinoma
PBT: Proton beam therapy
PVTT: Portal vein tumor thrombosis
GI: Gastrointestinal
CR: Complete response
CT: Computed tomography
POD: Postoperative day
