# Supplementary material for: Successful two-stage operation for esophageal necrosis due to proton beam therapy followed by sorafenib in a case of large hepatocellular carcinoma
Source: Surg Case Rep. 2020 Jun 16;6:138. doi: 10.1186/s40792-020-00902-0 (PMC7297925; doi:10.1186/s40792-020-00902-0)
Supplement: Supplementary file 1 — Additional file 1. Firstly, the operation was started from the normal anatomical region(a) and then the dissection line was connected to the fibrotic region(b), confirming the adjacent normal anatomy with magnified view(c). [file 40792_2020_902_MOESM1_ESM.pptx]

## Slide 1
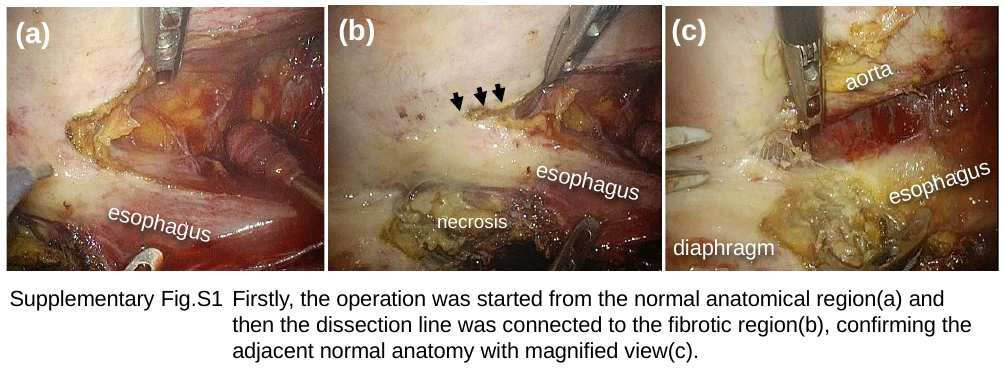

(c)
(b)
(a)
aorta
esophagus
esophagus
esophagus
Firstly, the operation was started from the normal anatomical region(a) and　then the dissection line was connected to the fibrotic region(b), confirming the adjacent normal anatomy with magnified view(c).
Supplementary Fig.S1
necrosis
diaphragm
